# Case Report: Recurrent retroperitoneal dedifferentiated liposarcoma after surgery

**DOI:** 10.3389/fonc.2026.1961498

**Published:** 2026-09-28

**Authors:** Deng Hu, Yao-dong Wang, Shi-chun Jiang, Decai Wang, Ding-ce Sun

**Affiliations:** Department of Urology, Mianyang Central Hospital, School of Medicine, University of Electronic Science and Technology of China, Mianyang, Sichuan, China

**Keywords:** abemaciclib, CDK4/6 inhibitor, combination therapy, radiotherapy, retroperitoneal dedifferentiated liposarcoma

## Abstract

**Objective:**

This study aims to summarize a comprehensive therapeutic strategy for recurrent retroperitoneal dedifferentiated liposarcoma (DDLPS) and evaluate the potential value of combining targeted therapy with radiotherapy.

**Methods:**

We retrospectively reviewed the complete clinical course of a patient with recurrent retroperitoneal DDLPS, focusing on recurrence patterns following the initial R0 resection, secondary surgical management, molecular testing findings, targeted therapy, radiotherapy, and follow-up outcomes. Additionally, a relevant literature review was conducted to support the analysis.

**Results:**

The first recurrence, measuring 1.3 cm, occurred 21 months after the initial operation, with the lesion enlarging to 4.7 cm by month 28, leading to a second laparoscopic resection. A second recurrence was noted 3 months postoperatively. Based on MDM2/CDK4 amplification, abemaciclib was administered at a dosage of 150 mg twice daily, resulting in stable disease with less than 20% tumor growth. Radiotherapy (50 Gy/25 fractions) was subsequently added after 2 months of targeted therapy, leading to tumor shrinkage to 4.8 cm × 3.1 cm (a 21.3% reduction in longest diameter) and a marked reduction in solid components. Per RECIST 1.1 criteria, the best overall response was stable disease (SD), although the reduction in tumor density suggested a clinically favorable response.

**Conclusions:**

Retroperitoneal DDLPS is characterized by a high recurrence rate. Complete surgical resection remains the cornerstone of treatment for localized disease. In recurrent cases, CDK4/6 inhibition guided by molecular profiling may provide effective disease control, and the addition of radiotherapy after targeted therapy appeared to confer an additive benefit in this patient. However, these findings are hypothesis-generating and require validation in future prospective studies.

## 1 Introduction

Dedifferentiated liposarcoma (DDLPS) is an aggressive subtype of liposarcoma characterized by invasive biological behavior. It most commonly arises in the retroperitoneum and is associated with a high risk of local recurrence and metastatic potential. DDLPS typically evolves from a well-differentiated liposarcoma (WDLPS) component that undergoes dedifferentiation into a non-lipogenic sarcoma (1–3). The majority of DDLPS tumors harbor gene amplifications in the 12q13–15 region, most notably MDM2 and CDK4 overexpression, which serve as useful diagnostic molecular markers (4, 5). The clinical and molecular heterogeneity of liposarcoma has been well documented (6), underscoring the need for subtype-specific management strategies. Surgery remains the cornerstone of treatment; however, radiotherapy, chemotherapy, and targeted therapies are increasingly employed as adjunctive modalities. Recent evidence suggests that radiotherapy may improve local control and potentially exert additive effects when combined with targeted agents. Given the rarity of cases presenting with early re-recurrence following a second surgery and subsequent treatment with a CDK4/6 inhibitor combined with radiotherapy, we report the management of a patient with multiply recurrent retroperitoneal DDLPS and discuss the implications of this combined approach.

## 2 Case presentation

A 61-year-old male patient was admitted to our hospital on August 11, 2024, with a diagnosis of recurrent right retroperitoneal dedifferentiated liposarcoma following prior surgery. Approximately two years earlier, he had undergone resection of a giant retroperitoneal tumor with right nephrectomy due to tumor invasion of the right kidney. According to the available pathology records, the primary tumor measured approximately 35 cm × 28 cm × 10 cm, was classified as FNCLCC grade 3, and was resected with R0 margins; the pathological diagnosis was dedifferentiated liposarcoma (7). During postoperative follow-up, a recurrent nodule of approximately 1.3 cm was detected in the right retroperitoneum at 21 months after the initial surgery; however, no intervention was undertaken at that time. At 28 months, abdominal MRI revealed that the lesion had enlarged to approximately 2.9 cm × 3.5 cm × 4.7 cm (**Figure 1A**), suggestive of local recurrence with marked interval growth.

**Figure 1:**
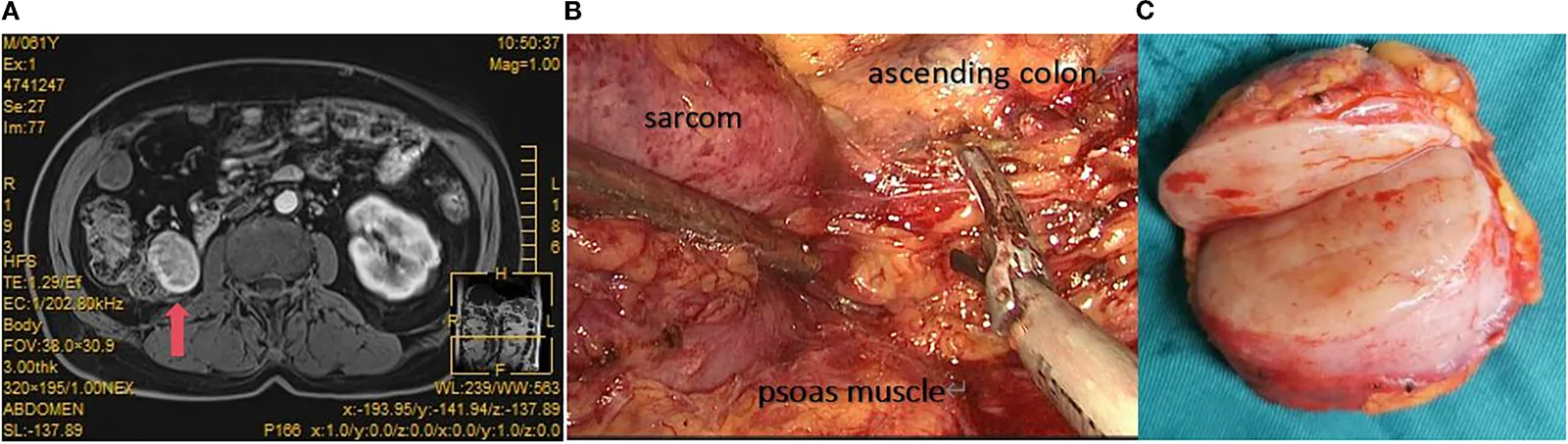
Preoperative MRI, intraoperative findings, and gross specimen of the second surgery for recurrent retroperitoneal dedifferentiated liposarcoma. **(A)** Preoperative MRI, red arrow indicates the tumor. **(B)** Intraoperative view. **(C)** Cut section of the gross specimen.

On August 15, 2024, the patient underwent laparoscopic resection of the recurrent tumor. Intraoperatively, an approximately 5-cm encapsulated mass was identified in the retroperitoneum (**Figure 1B**). The tumor was completely excised with R0 margins. Histopathological examination revealed spindle cell proliferation, myxoid stromal changes, scattered atypical cells, and lipoblasts. Immunohistochemistry showed MDM2 (partial positive) and CDK4 (positive), and FISH demonstrated MDM2 amplification (86%) and CDK4 amplification (82%), confirming recurrent DDLPS (**Figure 1C**). RB1/Rb status was not evaluated due to the unavailability of residual tumor tissue.

At 3 months postoperatively, CT revealed a new irregular mass (approximately 5.7 cm × 3.8 cm) in the right renal fossa (**Figure 2A**). The patient was started on abemaciclib 150 mg twice daily. A CT scan performed 2 months later showed a slight increase to 6.1 cm × 4.5 cm, which was classified as stable disease (SD) per RECIST 1.1 (**Figure 2B**). At month 2 of targeted therapy, local radiotherapy was administered (50 Gy/25 fractions). Three months after radiotherapy, imaging demonstrated tumor shrinkage to 4.8 cm × 3.1 cm with decreased solid component density (**Figure 2C**). Per RECIST 1.1, the longest diameter decreased from 6.1 cm to 4.8 cm (a 21.3% reduction), which did not meet the threshold for partial response (≥30% decrease) and was therefore classified as SD.

**Figure 2:**
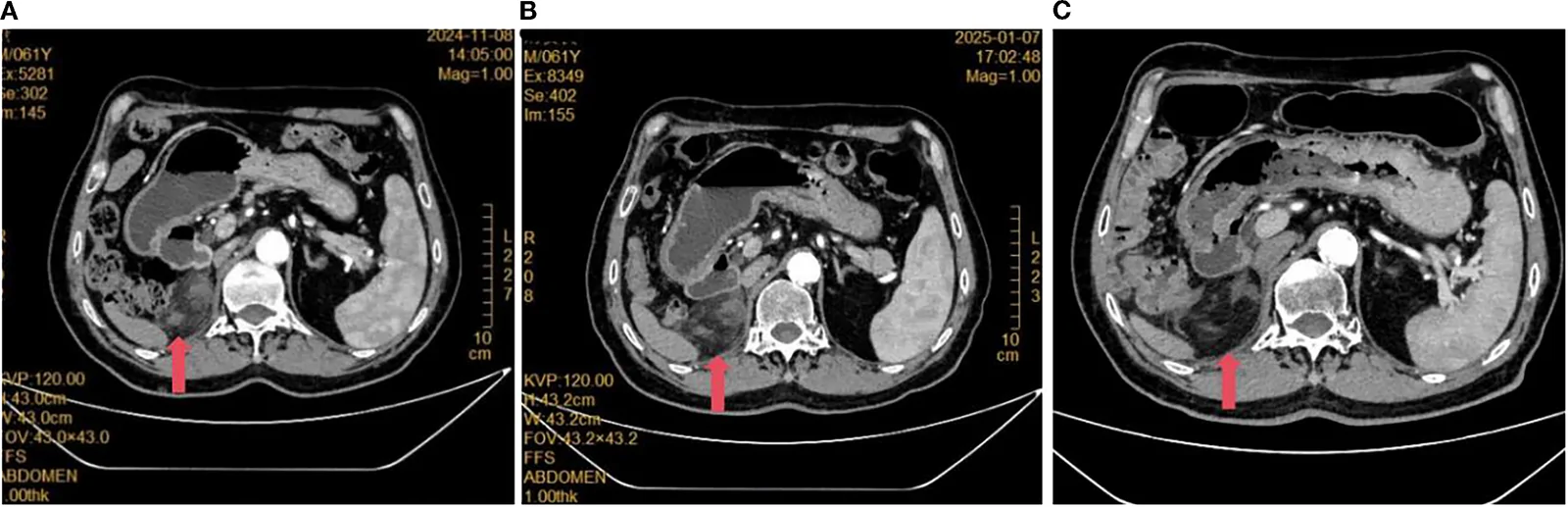
Serial abdominal CT images of the recurrent retroperitoneal DDLPS (red arrows indicate the tumor). **(A)** At 3 months after the second surgery, showing a new mass (5.7 cm × 3.8 cm) in the right renal fossa. **(B)** After 2 months of abemaciclib, showing minimal enlargement to 6.1 cm × 4.5 cm, classified as stable disease (SD) per RECIST 1.1. **(C)** At 3 months after radiotherapy, showing tumor shrinkage to 4.8 cm × 3.1 cm (a 21.3% reduction) with decreased solid component density, classified as SD by RECIST 1.1 (PR requires ≥30% decrease).

The patient developed grade 1 fatigue and grade 1 neutropenia (ANC nadir 1.4 × 10^9^/L) during the first two months of therapy, managed conservatively without dose reduction or interruption. No diarrhea, grade ≥2 hematologic toxicity, or liver enzyme elevation was observed. Radiotherapy-related toxicity was limited to grade 1 nausea. The patient continues on abemaciclib and remains clinically stable with no evidence of progression. At the most recent follow-up in July 2026, imaging showed no significant change, indicating sustained disease control. A detailed clinical timeline is presented in **Figure 3**.

**Figure 3:**
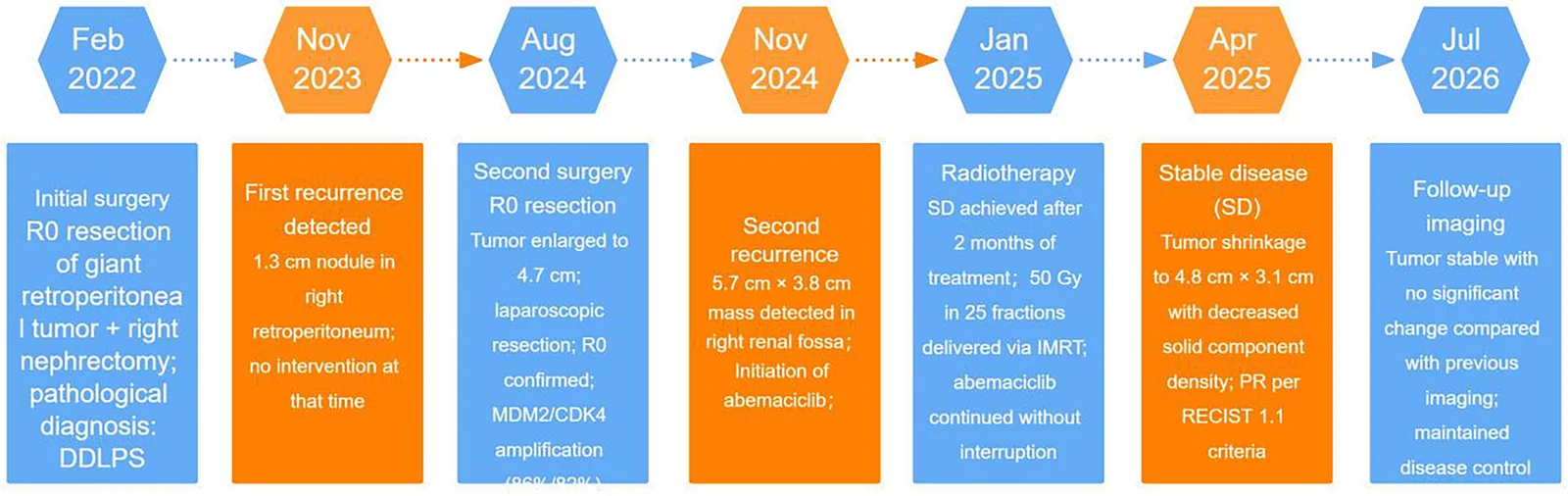
Clinical timeline of the patient’s disease course. Key events are displayed chronologically: initial surgery (R0 with right nephrectomy), first recurrence detection, second laparoscopic resection (R0), molecular testing (MDM2/CDK4 amplification), initiation of abemaciclib, radiotherapy (50 Gy/25 fractions), and response assessment (SD per RECIST 1.1 with tumor shrinkage). SD, stable disease; RECIST 1.1, Response Evaluation Criteria in Solid Tumors version 1.1.

Written informed consent was obtained from the patient for the publication of this case report and any accompanying images.

## 3 Discussion

For localized or resectable DDLPS, complete surgical resection (R0) remains the cornerstone of treatment and the only potentially curative approach (8, 9). However, even with R0 resection, the 5-year local recurrence rate for large abdominal liposarcomas has been reported to be as high as 85% (10). The fundamental reasons for surgical failure include the aggressive biological behavior of the tumor—particularly the dedifferentiated component—and its propensity for microscopic infiltration, even when a “pseudocapsule” appears intact on imaging or during surgery. In our patient, the recurrent lesion appeared well-encapsulated at the second operation, yet re-recurrence occurred within 3 months, which strongly supports this notion. Moreover, multiple recurrences are prone to subtype shifting, and a higher proportion of DDLPS component is associated with increased risks of local relapse and distant metastasis (11). Our patient recurred twice within a short interval (21 months after the first surgery and only 3 months after the second), consistent with the aggressive natural history of DDLPS.

For resectable locally recurrent tumors, surgery remains the preferred treatment option (12). However, with each successive recurrence, surgical difficulty increases and the likelihood of achieving R0 resection decreases (13, 14). Clinical consensus guidelines suggest that for high-grade liposarcomas with short recurrence intervals (e.g., <1 year) or rapid growth (e.g., >1 cm/month), repeat surgery may not significantly prolong survival (15). Nevertheless, for recurrent lesions larger than 10 cm or those causing significant organ compression symptoms, surgery may still be considered for palliative symptom relief (16).

For advanced unresectable or metastatic DDLPS, anthracycline-based regimens remain the standard first-line systemic chemotherapy option (17); however, response rates are low and prognosis remains poor, with a 5-year survival rate of only approximately 30% (18). Some reviews have suggested that preoperative radiotherapy may reduce local/abdominal recurrence in low-grade liposarcomas, but clear evidence of improved survival is lacking, and it is associated with higher perioperative serious adverse events (19). A systematic review and clinical practice guidelines have further defined the role of radiation therapy in primary retroperitoneal sarcoma (20), and retrospective studies have suggested that postoperative adjuvant radiotherapy may confer a survival benefit for retroperitoneal liposarcomas larger than 10 cm with poor or undifferentiated histology (21). Additionally, radiotherapy has been shown to modulate the immune microenvironment of DDLPS, with 63.6% of DDLPS patients showing increased tumor-infiltrating lymphocyte (TIL) scores after radiotherapy (22), providing a rationale for combining radiotherapy with targeted therapy.In our patient, after SD with gradual enlargement on abemaciclib, the addition of radiotherapy resulted in tumor shrinkage with reduced solid components. Although the 21.3% reduction did not meet RECIST criteria for PR, the lesion showed notable qualitative improvement. However, the individual contributions of each modality cannot be clearly distinguished, as radiotherapy was added after SD had been achieved. This limitation precludes any firm conclusion regarding synergy, although the temporal association supports the hypothesis that the combination may have conferred additional benefit, warranting further investigation.

Given that DDLPS almost universally harbors CDK4 amplification, frequently in conjunction with MDM2 amplification, targeting the CDK4/6 pathway represents a highly promising therapeutic strategy. CDK4/6 are key regulators of the G1–S cell cycle transition, and their overexpression drives aberrant tumor cell proliferation. The NCCN guidelines list palbociclib as a treatment option for advanced DDLPS, particularly in later-line settings (23), based on clinical studies demonstrating PFS benefits (24). However, CDK4 amplification alone is not a validated predictive biomarker for response, and its presence does not guarantee clinical benefit (25). In this case, the decision to use abemaciclib was based on the biological rationale of pathway activation combined with published clinical data. Palbociclib specifically inhibits CDK4/6, preventing Rb phosphorylation and arresting the cell cycle at G1. Abemaciclib is another CDK4/6 inhibitor with higher inhibitory potency and more sustained target inhibition, partly attributable to its distinct pharmacokinetic properties, including a longer half-life and greater tissue penetration (26). A pivotal single-arm phase II study (NCT02846987) demonstrated clinically meaningful PFS and disease control rates with acceptable tolerability (26). Based on these data, our patient was started on abemaciclib (150 mg twice daily) after the second recurrence. Initial imaging classified the lesion as SD per RECIST 1.1, suggesting therapeutic activity of CDK4/6 inhibition in this molecularly selected patient, although this remains hypothesis-generating. Immunotherapy was not pursued, as PD-L1, TMB, and MSI testing were not available (27).

The management of our patient illustrates the practical application of precision medicine in DDLPS, where molecular profiling (28) guided the selection of abemaciclib based on MDM2/CDK4 amplification. After initial disease control with the lesion classified as SD, gradual enlargement prompted the addition of radiotherapy, which resulted in tumor shrinkage and suggested a potential additive effect warranting further investigation. The proposed mechanism—CDK4/6 inhibition impairing DNA repair and enhancing radiosensitivity—is supported by preclinical literature but was not directly demonstrated in this case. This combined approach may represent a treatment option for patients with locally progressive and unresectable sarcoma.

In conclusion, DDLPS is a high-grade liposarcoma subtype with high rates of recurrence and metastasis. R0 resection remains the key to curative treatment, particularly for retroperitoneal lesions. For unresectable or advanced disease, anthracycline-based chemotherapy has limited efficacy, while targeting the CDK4/6 pathway has emerged as a significant breakthrough. Radiotherapy plays an important role in local disease control and may exert additive effects when combined with targeted therapy, although this requires confirmation in larger prospective studies. In our patient, molecular-guided abemaciclib therapy followed by radiotherapy upon progression successfully controlled the tumor, suggesting a potential treatment strategy. However, these findings are hypothesis-generating and require validation. Future prospective studies with longer follow-up are needed to confirm the durability and survival benefit of this approach.

We acknowledge several limitations in this case report. First, detailed pathological parameters of the primary tumor, such as mitotic count and necrosis percentage, were not available for re-evaluation, as archived specimens from the initial surgery were not retrievable. Second, RB1/Rb status was not evaluated due to the lack of residual tumor tissue for additional molecular testing, precluding direct assessment of CDK4/6 inhibitor sensitivity. Third, although the patient has been followed for approximately 15 months after radiotherapy and 20 months after initiation of abemaciclib with sustained disease control, longer follow-up is still required to confirm the durability of the observed response. Fourth, as a single case report, our findings are hypothesis-generating and require validation in future studies. Despite these limitations, we believe this case provides valuable real-world insights into the multidisciplinary management of recurrent retroperitoneal DDLPS, particularly regarding the potential additive effect of radiotherapy after CDK4/6 inhibition.

We acknowledge several limitations in this case report. First, detailed pathological parameters of the primary tumor, such as mitotic count and necrosis percentage, were not available for re-evaluation, as archived specimens from the initial surgery were not retrievable. Second, RB1/Rb status was not evaluated due to the lack of residual tumor tissue for additional molecular testing, precluding direct assessment of CDK4/6 inhibitor sensitivity. Third, although the patient has been followed for approximately 18 months after radiotherapy and 20 months after initiation of abemaciclib with sustained disease control, longer follow-up is still required to confirm the durability of the observed response. Fourth, as a single case report, our findings are hypothesis-generating and require validation in future studies. Despite these limitations, we believe this case provides valuable real-world insights into the multidisciplinary management of recurrent retroperitoneal DDLPS, particularly regarding the potential additive effect of radiotherapy after CDK4/6 inhibition.

This case report was prepared in accordance with the CARE (CAse REport) guidelines (29). All required elements, including a clinical timeline, de-identified patient information, diagnostic and therapeutic details, follow-up outcomes, strengths and limitations, and patient perspective with informed consent, have been included. The completed CARE checklist is available as a supplementary file.

## 4 Patient perspective

The patient reported that he had been in good health before the diagnosis, which came as an unexpected shock. He experienced anxiety during the first recurrence but decided to proceed with abemaciclib after detailed discussions with the oncology team. He tolerated the medication well, with only mild fatigue and no major side effects. He found radiotherapy smooth and the staff supportive, and was encouraged by imaging results showing tumor shrinkage. He continues regular follow-up and hopes sharing his experience may help other patients.

## Data Availability

The raw data supporting the conclusions of this article will be made available by the authors, without undue reservation.
